# P18 Retrospective study of chlamydia contacts and management

**DOI:** 10.1093/jacamr/dlae217.022

**Published:** 2025-01-23

**Authors:** Zahra Ahmed, Jenny Kent, Angela Bailey

**Affiliations:** NHS Buckinghamshire, UK; NHS Buckinghamshire, UK; NHS Buckinghamshire, UK

## Abstract

**Background:**

Antimicrobial resistance (AMR) poses a significant public health threat by reducing the effectiveness of established treatments for bacterial infections. Unnecessary antibiotic use can lead to an inability to treat common infections, increase healthcare costs, and worsen morbidity and mortality. In the context of chlamydia management, treating contacts prophylactically who are not infected may not provide clinical benefit, yet exposes them to antibiotics. The initial reason for treating contacts is to prevent associated complications and decrease transmission in the population. Antibiotics within the context of chlamydia treatment have a 95% effectiveness rate as a first-time therapy. A study using an *in vitro* model of doxycycline as prophylaxis found that this use of antibiotics could directly contribute to the increase of antibiotic resistance in already difficult-to-treat species. Therefore, healthcare professionals working in sexual health should be aware of the risk and benefits of treating contacts and should implement appropriate antibiotic stewardship. The current Buckinghamshire Sexual Health and Wellbeing (BSHAW) trust policy is to treat chlamydia contacts who have had sex with index cases <2 weeks ago. If >2 weeks ago, then management should be based on symptoms and patient preference. This policy is in line with 2015 UK national guidelines based on BASHH recommendations that ‘patients should be treated if concerned about sexual exposure within 2 weeks’.

**Objectives:**

Assess the proportion of chlamydia contacts who were treated with doxycycline when their last sexual encounter with the index case was >2 weeks. Determine the percentage of these contacts who tested positive for chlamydia.

**Methods:**

A retrospective case note review of 77 chlamydia contact notes across BSHAW service between May and July 2024. All patients presented independently as chlamydia contacts. Diagnoses of chlamydia were confirmed by either first pass urine, high vaginal, rectal or pharyngeal swabs.

**Results:**

Across 3 months, 39 contacts presented within a 2 week window and 38 contacts presenting >2 week window. The average time for contacts to attend clinic independently was 15.03 days post-index case. All (100%) of the 39 contacts <2 week window were treated with doxycycline. Of these patients, 82% were asymptomatic, and 46% had a positive diagnostic result. The remaining 18% of the contacts were symptomatic with 58% testing positive. Of the 38 contacts, 74% presented a >2 week window, and 71% were asymptomatic. 63% were treated, of which 17% had a positive result. All symptomatic patients were treated, with 54% testing positive. Of the asymptomatic contacts, 82% were overtreated. 3% of the cohort were not treated despite being positive.

**Conclusions:**

As a department, we are overtreating chlamydia contacts >2 weeks from index case. Chlamydia contacts presenting >2 weeks from index case should not be given doxycycline unless tests are positive, or the contact has symptoms. A ‘test and wait’ process should be implemented to reduce unnecessary antibiotic prescriptions.

